# Supplementary material for: Single‐cell transcriptomics reveals immune response of intestinal cell types to viral infection
Source: Mol Syst Biol. 2021 Jul 26;17(7):e9833. doi: 10.15252/msb.20209833 (PMC8311733; doi:10.15252/msb.20209833)
Supplement: Supplementary file 1 — Appendix [file MSB-17-e9833-s004.pdf]

Table of contents

Appendix Figure S1: General information of single-cell RNA-seq samples from small intestine
Appendix Figure S2: Clustering and annotation of ileum biopsies
Appendix Figure S3: Clustering and annotation of ileum organoids
Appendix Figure S4: Additional information for Hiplex experiments
Appendix Figure S5: Astrovirus expression per cell type.
Appendix Figure S6: Multiplex in situ RNA hybridization visualizes induction of immune signature in different cell types upon infection.
Appendix Figure S7: Quantification of multiplex in situ RNA hybridization fluorescence from mock-treated and HAstV infected organoids
Appendix Figure S8: Comparison between live and fixed and FACS sorted and non-sorted single-cell RNA-seq samples from intestinal organoids
Appendix Table S1. Organoids culturing media.
Appendix Table S2. Primers used for qPCR.
Appendix Table S3. The RNAscope probes used for the multiplex RNA FISH on 3D organoids cryosections.
Appendix Table S4. The RNAscope probes used for the multiplex RNA FISH on 2D organoids.

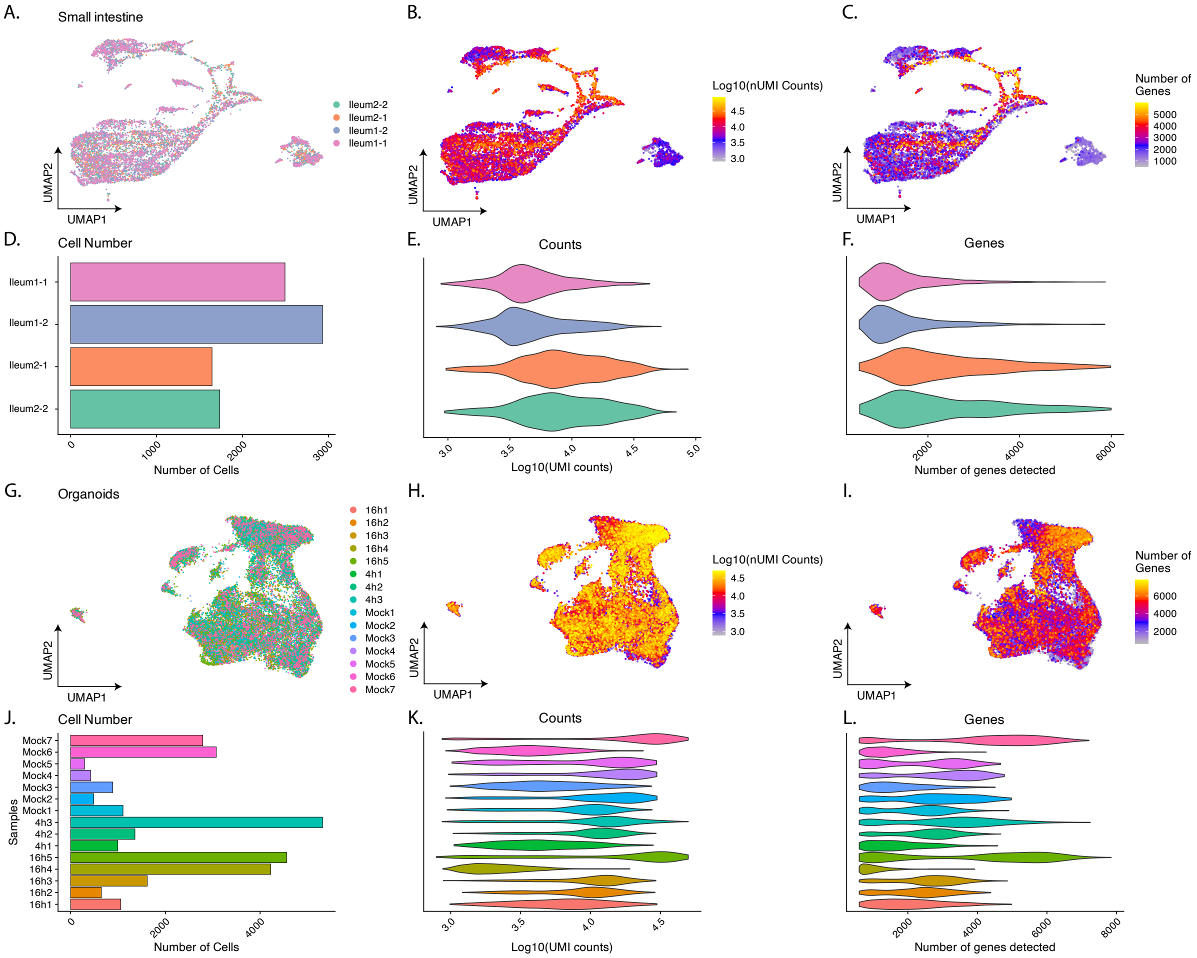

Appendix Figure S1. General information of single-cell RNA-seq samples from small intestine Data from. **A-F**. tissue and **G-L**. organoids. Uniform manifold approximation and projection (UMAP) embedding of single-cell RNA-Seq data from human ileum biopsies colored by sample **A.** and **G.**, Number of UMI couts **B.** and **H.** and genes **C.** and **I.** Statistics of the single-cell RNA-seq datasets after quality filters are also shown as Violin plot for every sample, Number of cells **D.** and **J.**, total UMI counts **E.** and **K.** and detected genes **F.** and **L.**

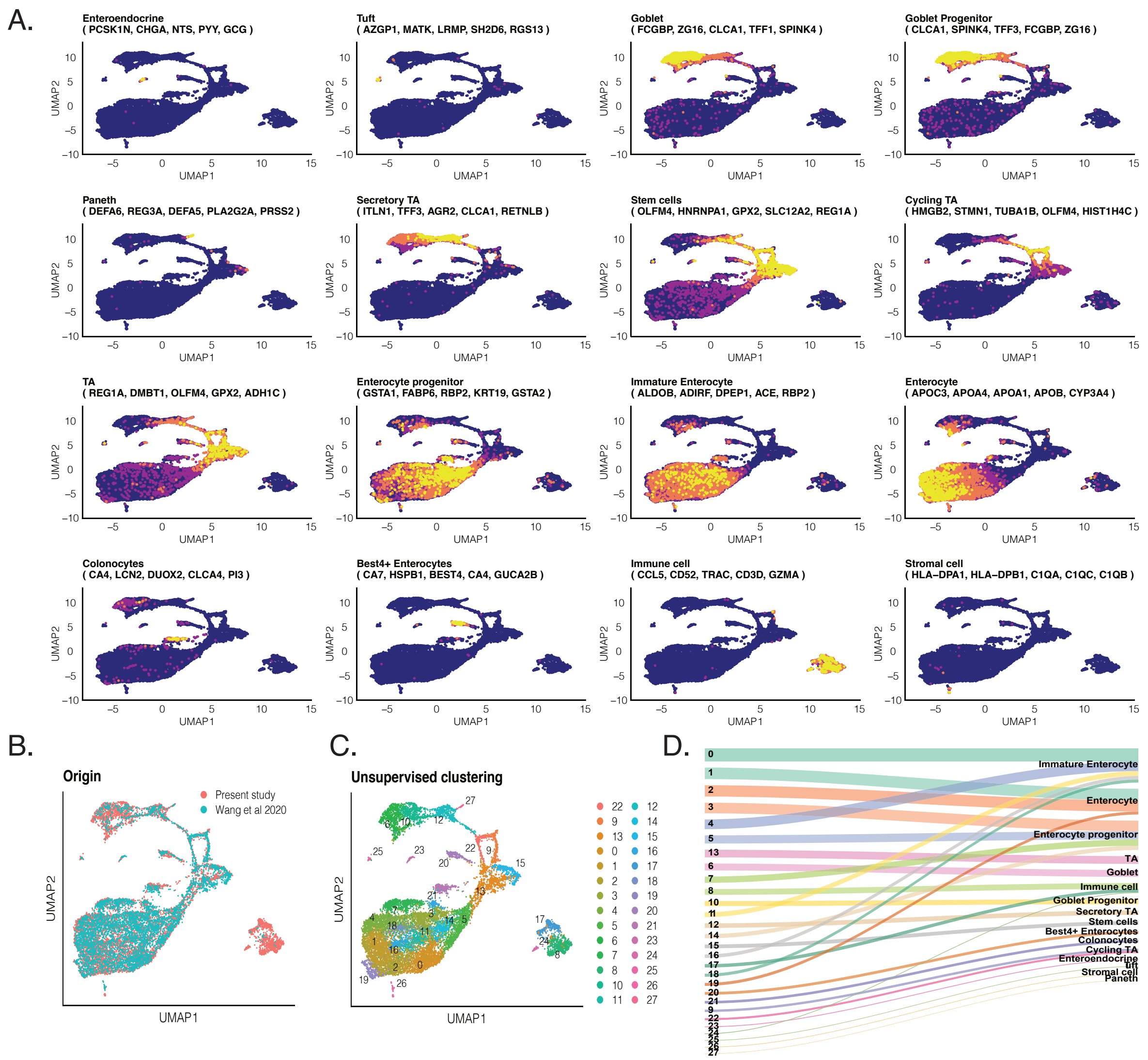

Appendix Figure S2. Clustering and annotation of ileum biopsies. **A**. Uniform manifold approximation and projection (UMAP) embedding of single-cell RNA-Seq data from human ileum biopsies colored by average expression of top cell type marker, **B**. Study of the samples and **C**. Unsupervised clustering. **D.** Mapping between unsupervised clustering and ileum cell types.

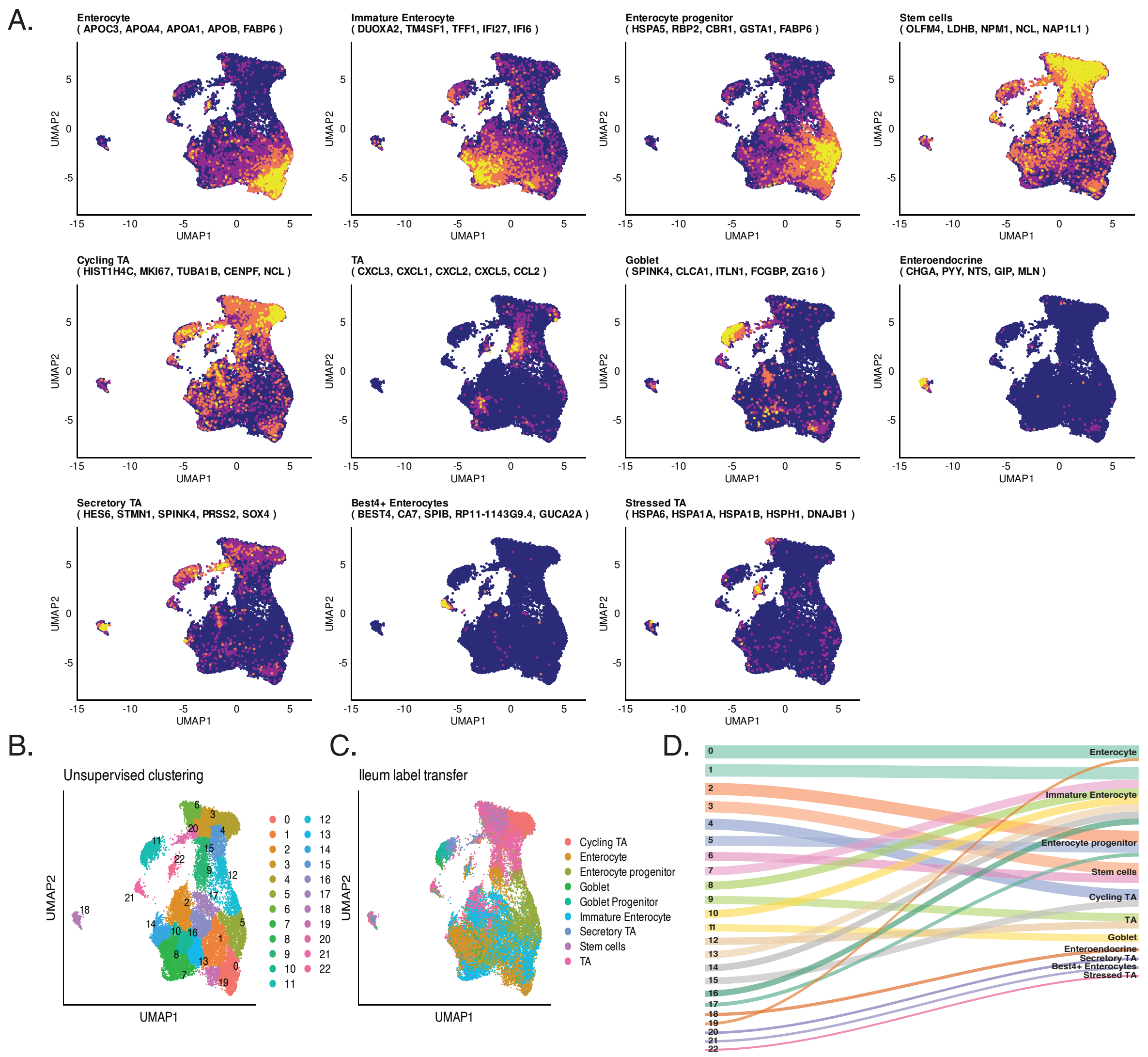

Appendix Figure S3. Clustering and annotation of ileum organoids. A. Uniform manifold approximation and projection (UMAP) embedding of single-cell RNA-Seq data from human ileum biopsies colored by average expression of top cell type marker, **B**. Unsupervised clustering and **C**. Label transfer from the ileum biopsies. **E.** Mapping between unsupervised clustering and organoids cell types.

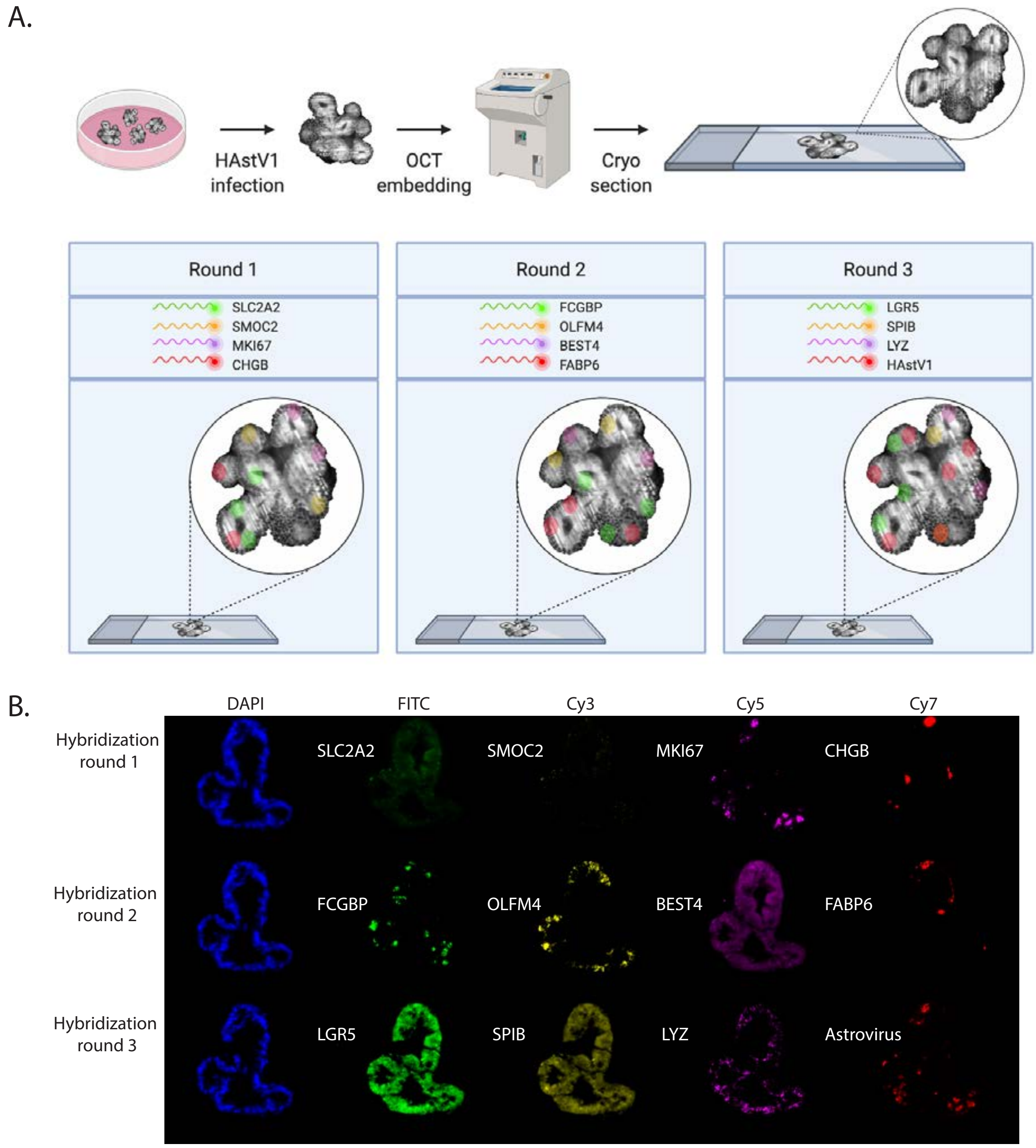

Appendix Figure S4. Additional information for Hiplex experiments. Representative FISH images of all 12 detected genes for cryosection of intestinal organoids. Splitted by hybridization round and colored by the channel used.

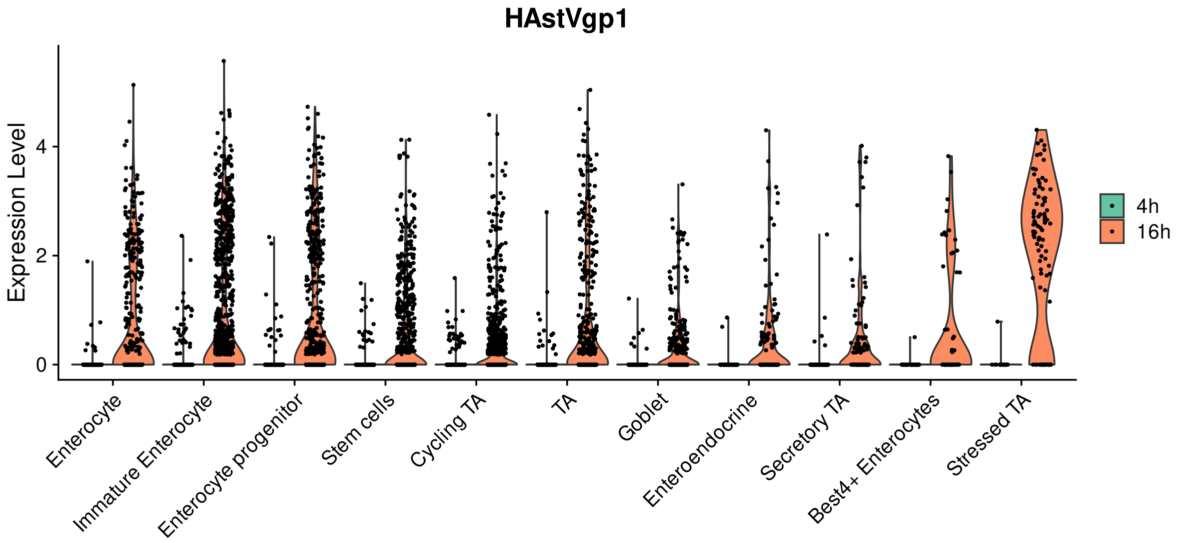

Appendix Figure S5: Astrovirus expression per cell type. Normalized expression levels of HAstVgp1 across each cell type for 4h and 16h pi.

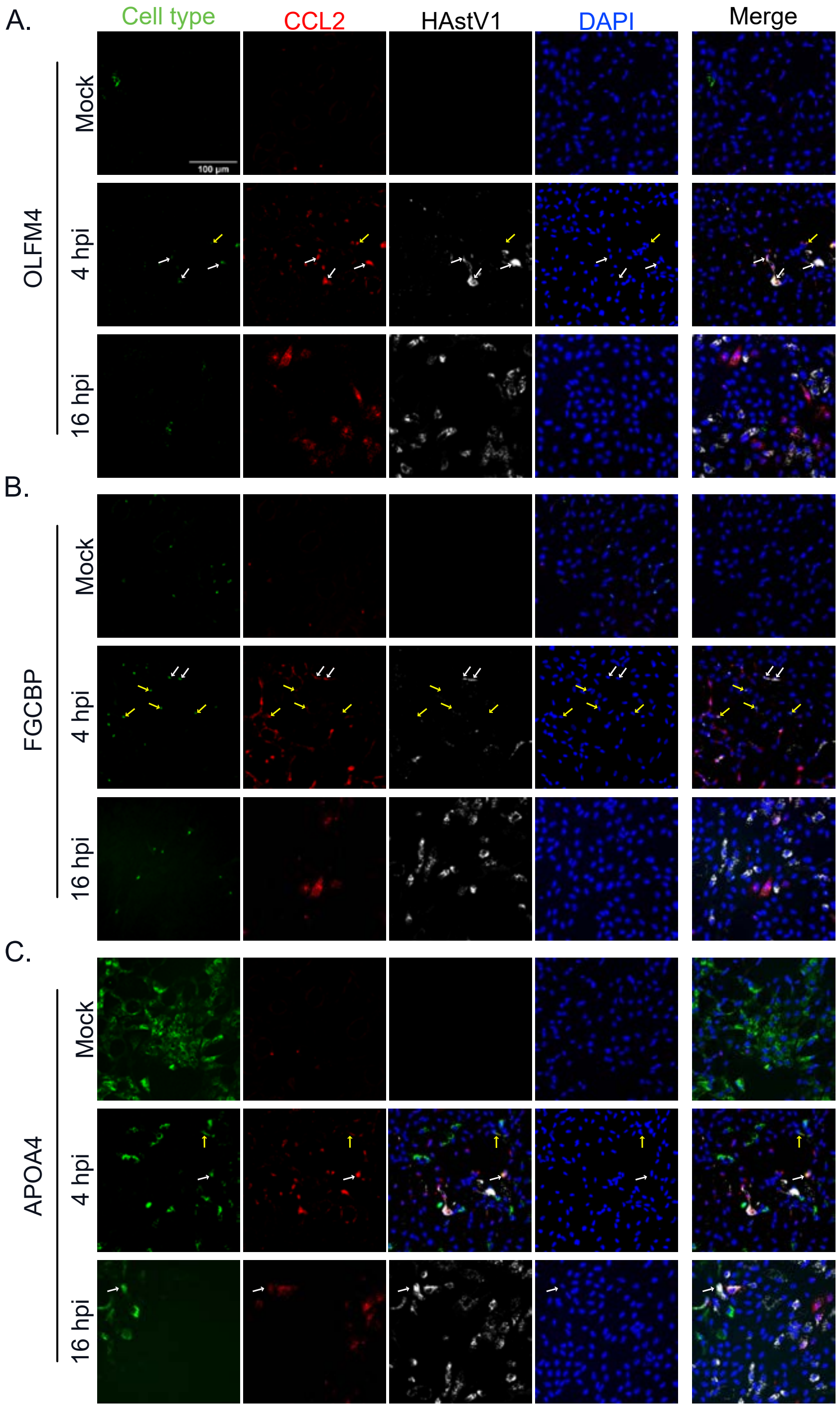

Supplementary Figure S6. Multiplex in situ RNA hybridization visualizes induction of immune signature in different cell types upon infection. A-C. One image per condition (the same as for Figure 5) was used to show the multiplex in situ RNA FISH. A representative region of the image was chosen for each cell lineage marker independently and cropped in order to have a zoom-in of a specific area for better visualization. The representative panels show the cell lineage markers (green), HAstV1 infection (white), and CCL2 gene expression (red) of mock-treated and infected organoids. DAPI is in blue. Yellow arrows show co-localization of cell lineage markers with CCL2 gene expression and white arrows show co-localization of cell lineage marker and CCL2 with HAstV signal. Scale bar 100µm.

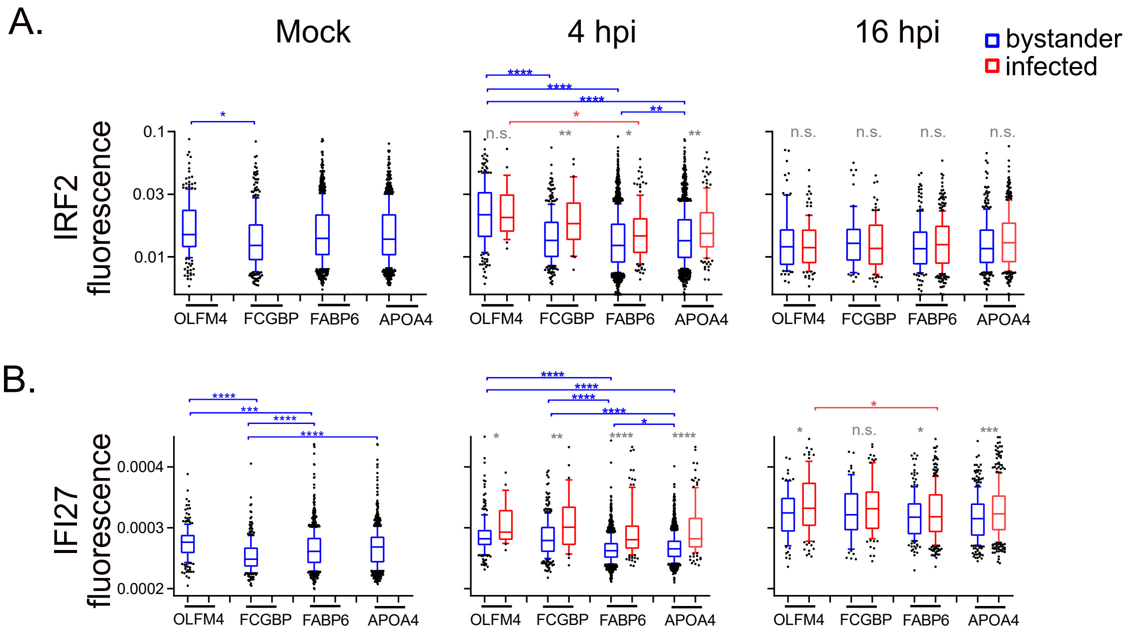

Supplementary Figure S7. Quantification of multiplex in situ RNA hybridization fluorescence from mock-treated and HAstV infected organoids. A-B. Fluorescence intensity (arbitrary units) of innate immune marker expression IRF2 and IFI27 in stem cells (OLFM4 positive), goblet cells (FCGBP positive), enterocyte lineage cells (FABP6 positive) and mature enterocytes (APOA4 positive). Bystander cells are in blue and infected cells in red. 10-90 percentile box plots, each dot represents one cell. Blue statistics show comparison of bystander cells between cell lineages and red statistics show comparison of infected cells between cell lineages. Ordinary one-way ANOVA and Tukey's multiple comparisons test was used. Grey statistics show comparison between infected and bystander cells within each cell lineage. Unpaired t test with Welch's correction was used. n.s non significant, * p<0.05, ** p<0.01, *** p<0.001, **** p<0.0001

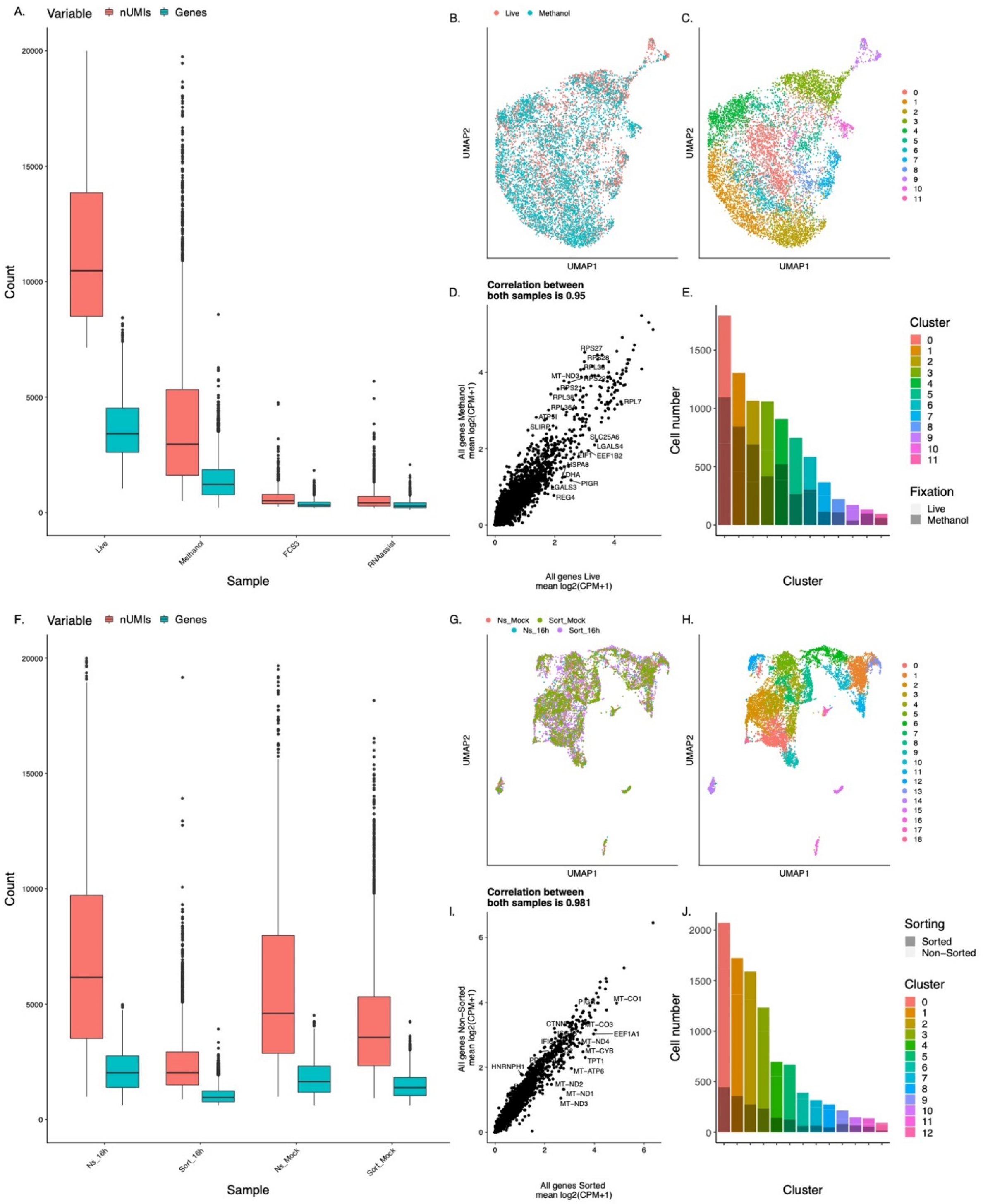

Appendix Figure S8. Comparison between live and fixed and FACS sorted and non-sorted single-cell RNA-seq samples from intestinal organoids. **A-E.** Fixed and live comparison. **F-J.** Sorted and non-sorted comparison. Boxplots showing the number of UMIs (blue) and genes (red) detected per cell using HIEs dissociated cells on **A.** Live, Methanol, FCS3 and RNAassist Fixed samples. **F.** Samples sorted and non-sorted at mock and 16hp.i **B and G**. UMAP representation colored by sample. **C and H.** UMAP colored by distinct cell clusters, found using unsupervised clustering. **D and I.** Pearson correlation calculated for each gene detected in both samples. **E and J.** Distribution of cells in each cluster according to the sample origin.

Appendix Table S1. Organoids culturing media.

Compound	*Final concentration*
**Basal media**	
Ad DMEM/F12<br>+GlutaMAX<br>+HEPES<br>+P/S	
Wnt3A	50% by volume
B27	1:50
N2	1:100
N-acetyl-cysteine	1mM
R-spondin	10% by volume
Noggin	100ng/mL
EGF	50ng/mL
Gastrin	10mM
Nicotinamide	10mM
A83-01	500nM
Sb202190	10uM
**Differentiation Media**	
Ad DMEM/F12<br>+GlutaMAX<br>+HEPES<br>+P/S	
B27	1:50
N2	1:100
N-acetyl-cysteine	1mM
R-spondin	5% by volume
Noggin	50ng/mL
EGF	50ng/mL
Gastrin	10mM
A83-01	500nM
Sb202190	10uM

Appendix Table S2. Primers used for qPCR.

Gene Name	Species	For seq	Rev seq
IFNl2/3	Human	gccacatagcccagttcaag	tgggagaggatatggtgcag
IFNb1	Human	gccgcattgaccatctat	gtctcattccagccagtg
HPRT1	Human	cctggcgtcgtgattagtgat	agacgttcagtcctgtccataa
HAstV1	Astrovirus	ccagtctcacagaagagcaac	cttgctagccatctcacttctt

Appendix Table S3. The RNAscope probes used for the multiplex RNA FISH on 3D organoids cryosections

Gene name	Role of the marker	Catalog number	Channel
SLC2A2	Mature enterocytes	NPRP-0000689	T1
SMOC2	Stem cells	522921	T2
MKI67	Cell cycle	591771	T3
CHGB	Enteroendocrine cells	567681	T4
FCGBP	Goblet cells	NPRP-0000691	T5
OLFM4	Stem-transit amplifying cells	311041	T6
BEST4	Best-M cells	481501	T7
FABP6	Enterocyte lineage	478701	T8
LGR5	Stem cells	311021	T9
SPIB	M cells	551431	T10
LYZ	Paneth cells	421441	T11
HAstVgp1	Astrovirus	NPR-0001571	T12

Appendix Table S3. The RNAscope probes used for the multiplex RNA FISH on 2D organoids.

Gene name	Role of the marker	Catalog number	Channel
APOA4	Mature enterocyte	857841	T1
STAT1		NPRP-0005454	T2
MKI67	Cell cycle	591771	T3
IFI27		NPRP-0005454	T4
FCGBP	Goblet cell	560181	T5
OLFM4	Stem-transit amplifying cells	311041	T6
CCL2		NPRP-0005454	T7
FABP6	Enterocyte lineage	478701	T8
ISG15	Infection	467741	T9
IRF2		NPRP-0005453	T10
IFNGR1		NPRP-0005454	T11
HAstVgp1	Astrovirus	805941	T12
