# Supplementary material for: Single‐cell transcriptomics reveals immune response of intestinal cell types to viral infection
Source: Mol Syst Biol. 2021 Jul 26;17(7):e9833. doi: 10.15252/msb.20209833 (PMC8311733; doi:10.15252/msb.20209833)
Supplement: Supplementary file 2 — Expanded View Figures PDF [file MSB-17-e9833-s005.pdf]

Expanded View Figures

Figure EV1. Interferons protect human intestinal organoids from HastV1 infection.

A Caco-2 cells were infected with HastV1. At indicated times, the virus was visualized by indirect immunofluorescence for HastV1 (red). Nuclei were stained with DAPI (blue). Scale bar 10 μm.
B Quantification of the number of HAstV1-infected cells from A.
C Caco-2 cells were infected with HAstV1. At indicated times, the replication of HAstV1 was assessed by the genome copy number over time using qRT–PCR.
D Caco-2 cells were infected with HAstV1. At indicated times, the intrinsic innate immune induction of type I (IFNβ1) and III (IFNλ) IFN was evaluated.
E Caco-2 cells were pre-treated for 24 h with 2,000 IU/ml of IFNβ1 or 300 ng/ml of IFNλ-3. Interferons were maintained during the course of infection and HastV1-infected cells were visualized with indirect immunofluorescence (left) and the number of HAStV1-infected cells was quantified (right). Scale bar 10 μm.
F Caco-2 cells were pre-treated for 24 h with 2,000 IU/ml of IFNβ1 or 300 ng/ml of IFNλ-3. Interferons were maintained during the course of infection and replication of HAstV1 was assessed by qRT–PCR for the genome copy number.
G Caco-2 cells, T84 wild-type, and T84 cells lacking both the type I and type III IFN receptors (dKO) were infected with HAstV1. 24 hpi HAstV1 genome replication was evaluated by qRT–PCR for the genome copy number.

Data information: A–G Three biological replicates were performed for each experiment. Representative immunofluorescence images are shown. Error bars indicate the standard deviation. Statistics are from unpaired *t*-test.

© 2021 The Authors

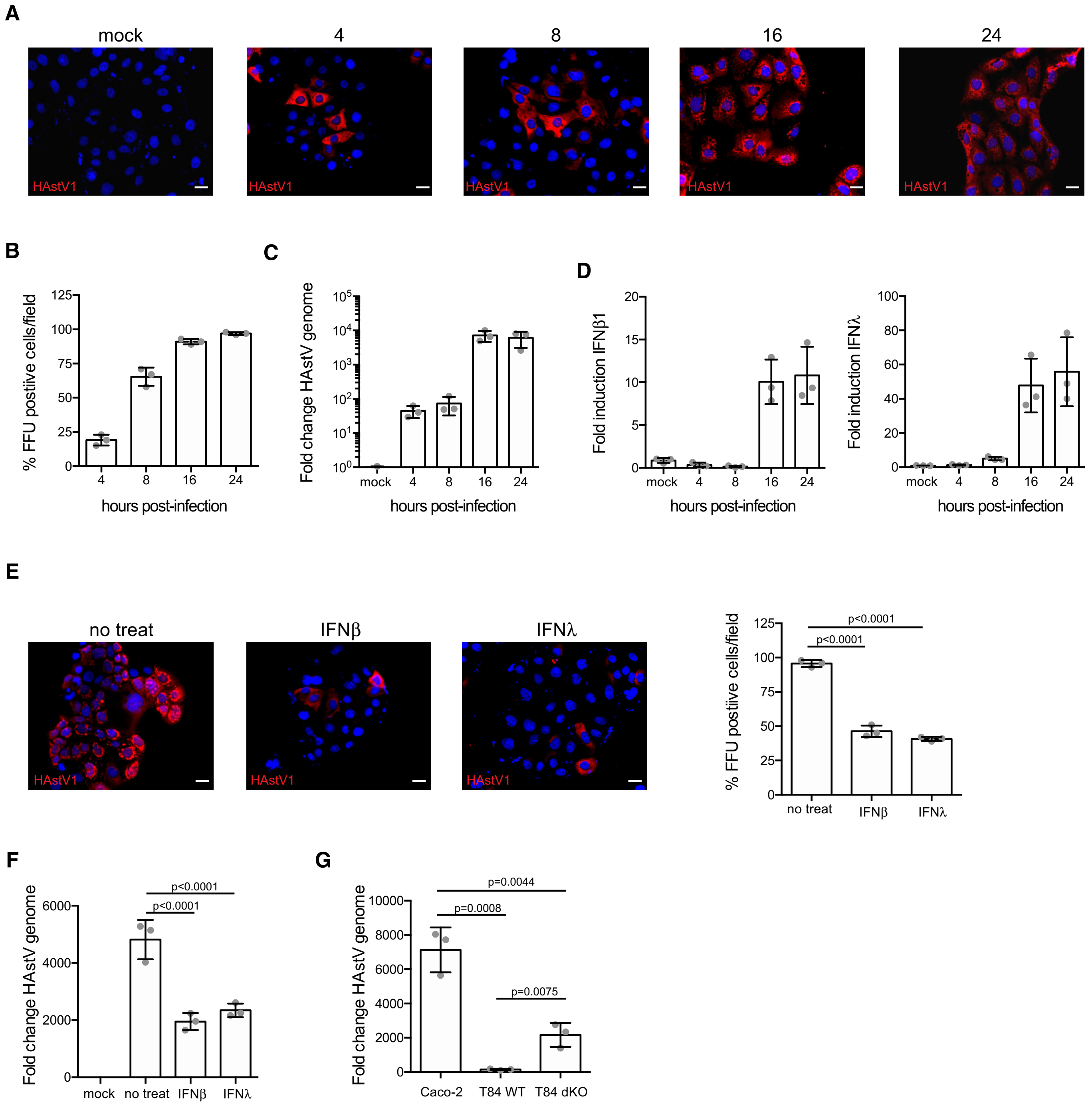

Figure EV1.

 © 2021 The Authors

Figure EV2. HAstV infection induces MKI67 expression in intestinal organoids.

A Representative images showing multiplex *in situ* RNA FISH of the proliferation marker MKI67 (green) and HAstV1 infection (red). DAPI is in blue. Scale bar 200 μm.

B Fluorescence intensity (arbitrary units, a.u.) of the proliferation marker MKI67 plotted against fluorescence intensity (a.u.) of HAstV1 RNA probe in mock-treated organoids and at 4 hpi and 16 hpi. Each dot represents a single cell, infected cells are in red and bystander cells in blue. Line shows a linear regression fit with a 95% confidence interval.

C Fluorescence intensity (a.u.) of the proliferation marker MKI67 expression in stem cells (OLFM4 positive), goblet cells (FCGBP positive), enterocyte lineage cells (FABP6 positive), and mature enterocytes (APOA4-positive). Infected cells are in red and bystander cells in blue. Floating bar (min and max), line is the mean. Blue statistics show comparison of bystander cells between cell lineages, and red statistics show comparison of infected cells between cell lineages. Ordinary one-way ANOVA and Tukey's multiple comparisons test were used. Gray statistics show comparison between infected and bystander cells within each cell lineage. Unpaired *t*-test with Welch's correction was used. n.s nonsignificant, **$P < 0.01$, ***$P < 0.001$, ****$P < 0.0001$.

© 2021 The Authors

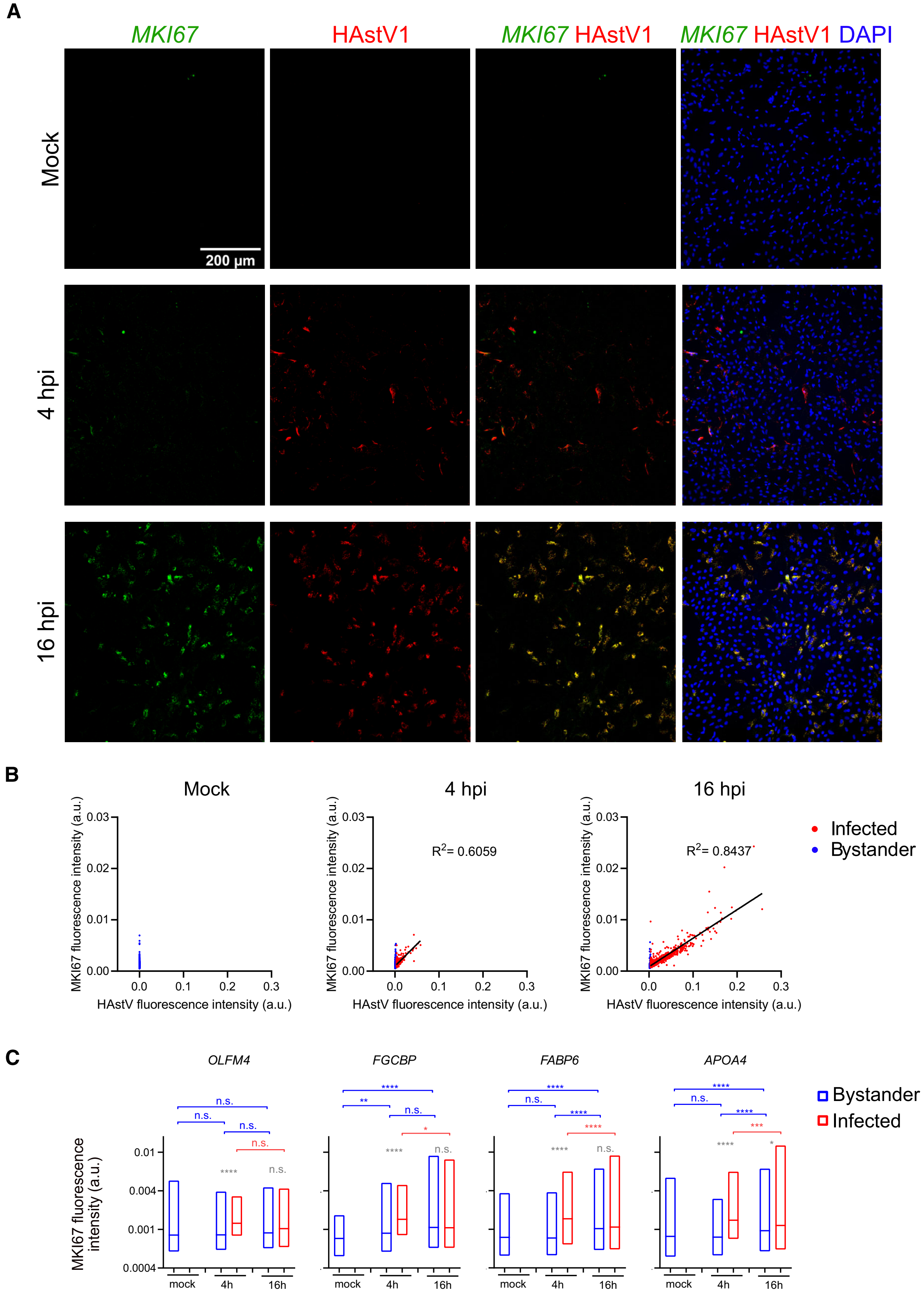

Figure EV2.

© 2021 The Authors

Figure EV3. Lineage-specific expression changes in mock versus 4 hpi and 16 hpi.

Volcano plots of genes that are differentially expressed in cells in one time point relative to the other, showing the statistical significance (−log10 adjusted P-value) versus log2 fold change and gene enrichment analysis results for significantly changing genes (FDR < 0.05)

A–D Enterocyte lineages.
E–H Transit-amplifying (TA) cells.
I–K Enteroendocrine cells. Selected genes are shown, all genes are reported in Dataset EV3.

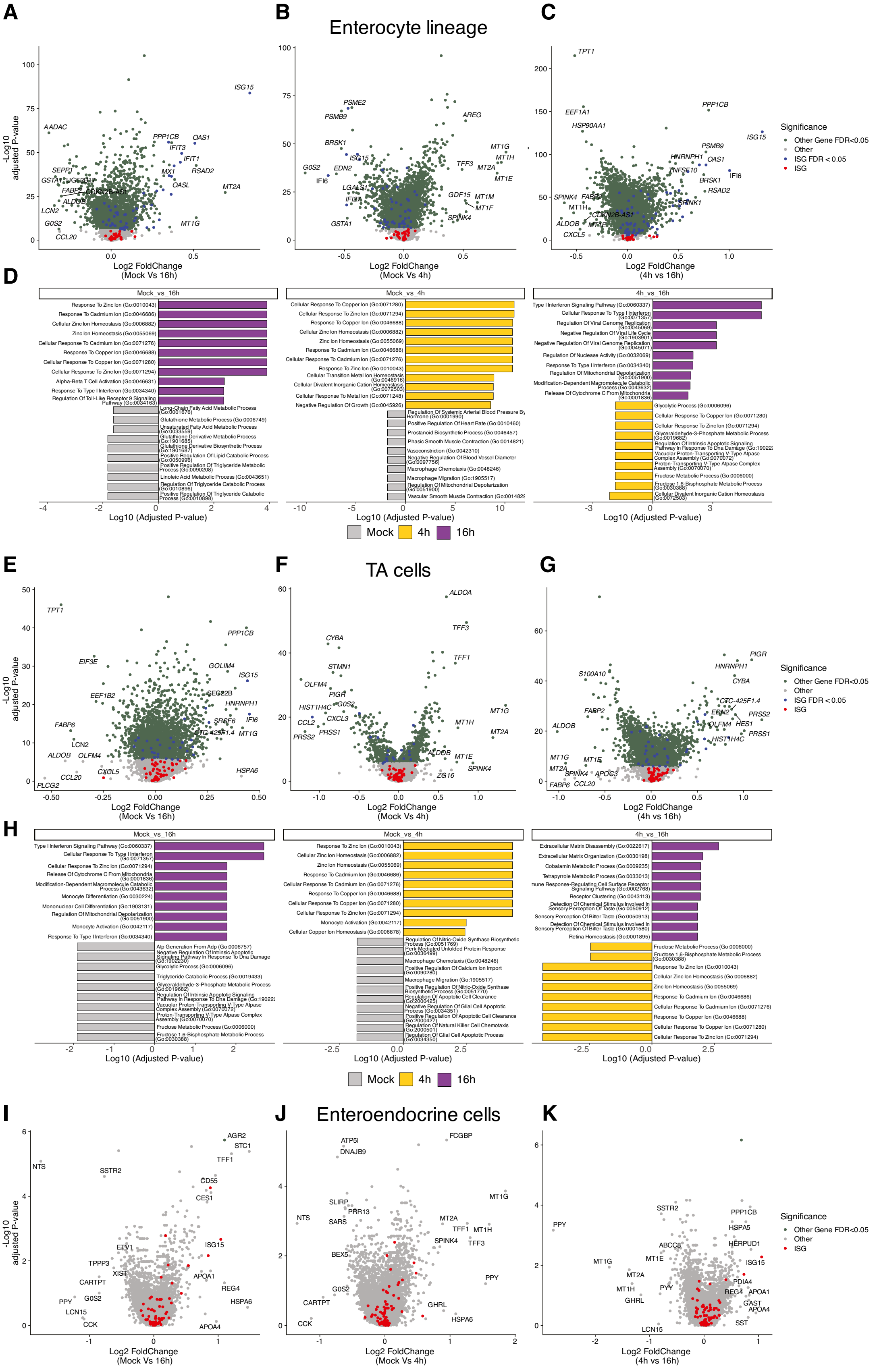

Figure EV3.

 © 2021 The Authors

Figure EV4. Lineage-specific expression changes in mock versus 4 hpi and 16 hpi.

Volcano plots of genes that are differentially expressed in cells in one time point relative to the other, showing the statistical significance (−log10 adjusted *P*-value) versus log2 fold change and gene enrichment analysis results for significantly changing genes (FDR < 0.05).

A–D Stem cells.
E–H Goblet cells
I–K Best4^{+} enterocytes. Selected genes are shown, all genes are reported in Dataset EV3.

© 2021 The Authors

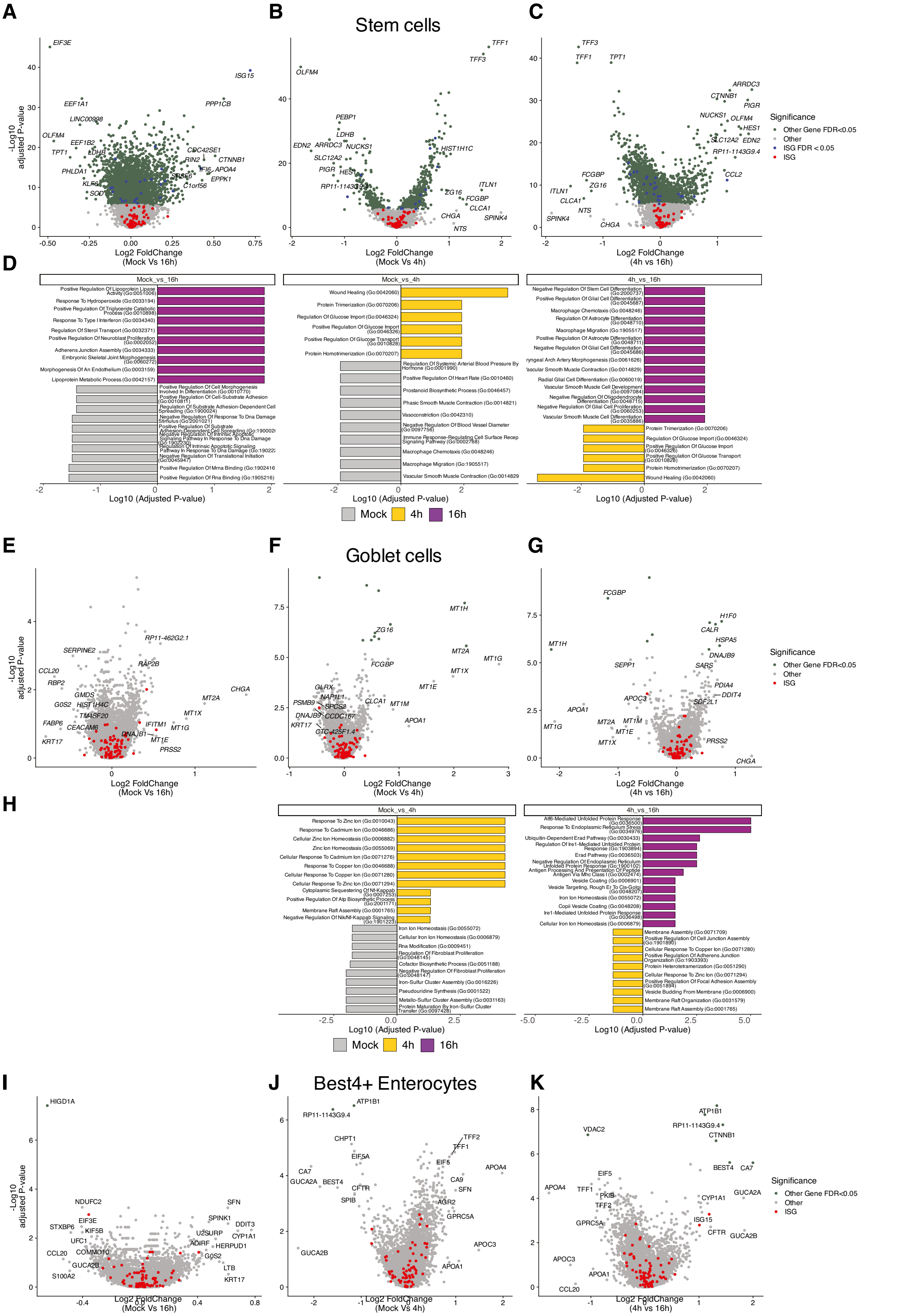

Figure EV4.

© 2021 The Authors

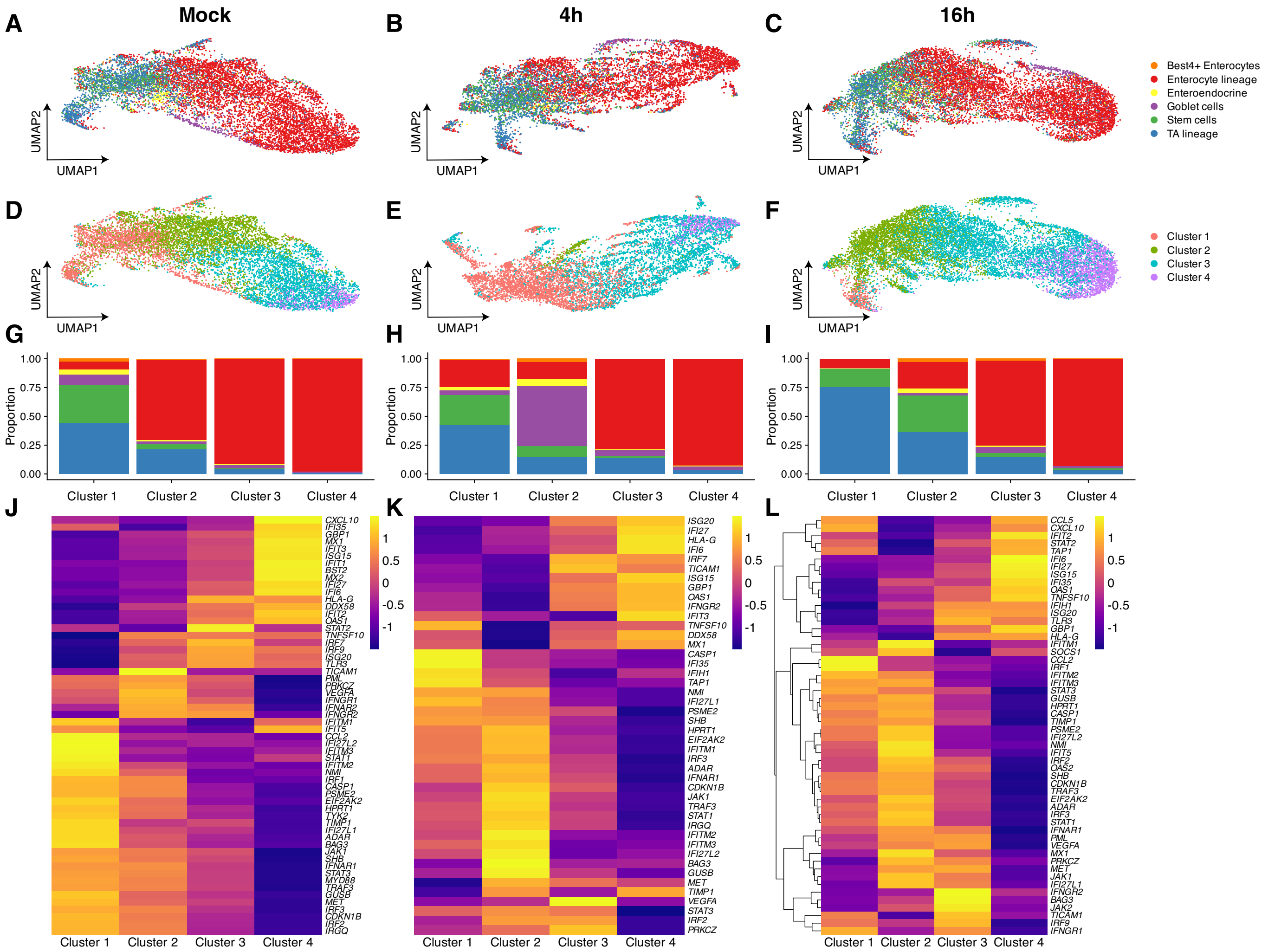

Figure EV5. Lineage-specific interferon patterns are conserved across conditions.

A–C UMAP embedding of scRNA-Seq data from human ileum-derived organoids based on the significantly changing ISGs for cells at A. Mock-infected, B. 4 hpi and C. 16 hpi.

D–F Unsupervised clustering of the UMAP data from A–C.

G–I The distribution of cell lineages and types in the clusters from D–F.

J–L A heatmap of differentially expressed ISGs across the clusters from D–F.

© 2021 The Authors
