# Supplementary figures and images for: Single‐cell transcriptomics reveals immune response of intestinal cell types to viral infection
Source: Mol Syst Biol. 2021 Jul 26;17(7):e9833. doi: 10.15252/msb.20209833 (PMC8311733; doi:10.15252/msb.20209833)

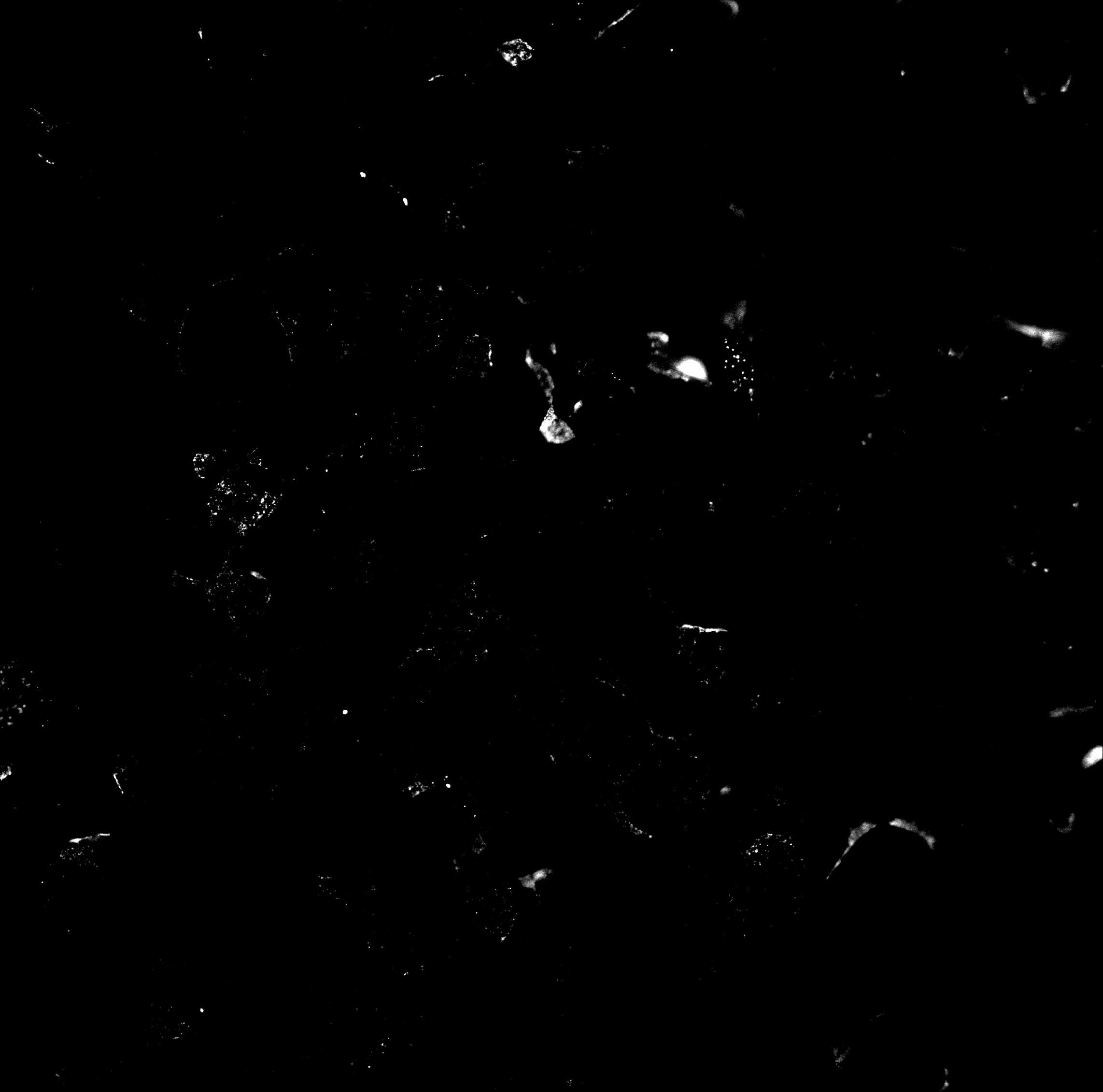

Supplement: Supplementary file 10 — Source Data for Figure 5 [file MSB-17-e9833-s001.zip › Figure 5/Figure 5A. 4hpi_FABP6(green) CCL2(red) DAPI(blue) AstV(white).tif]

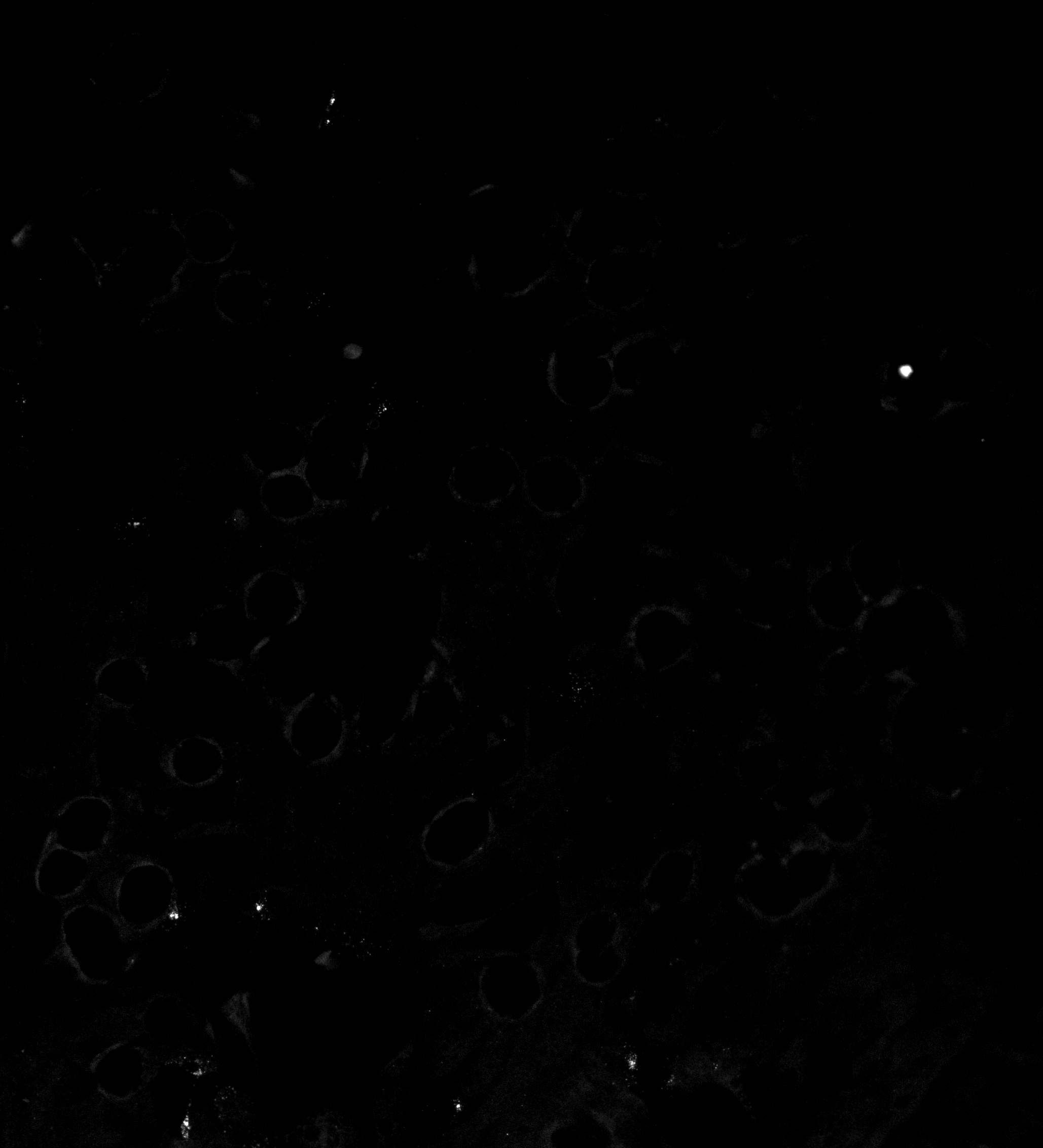

Supplement: Supplementary file 10 — Source Data for Figure 5 [file MSB-17-e9833-s001.zip › Figure 5/Figure 5A. Mock_ FABP6(green) CCL2(red) DAPI(blue) AstV(white).tif]

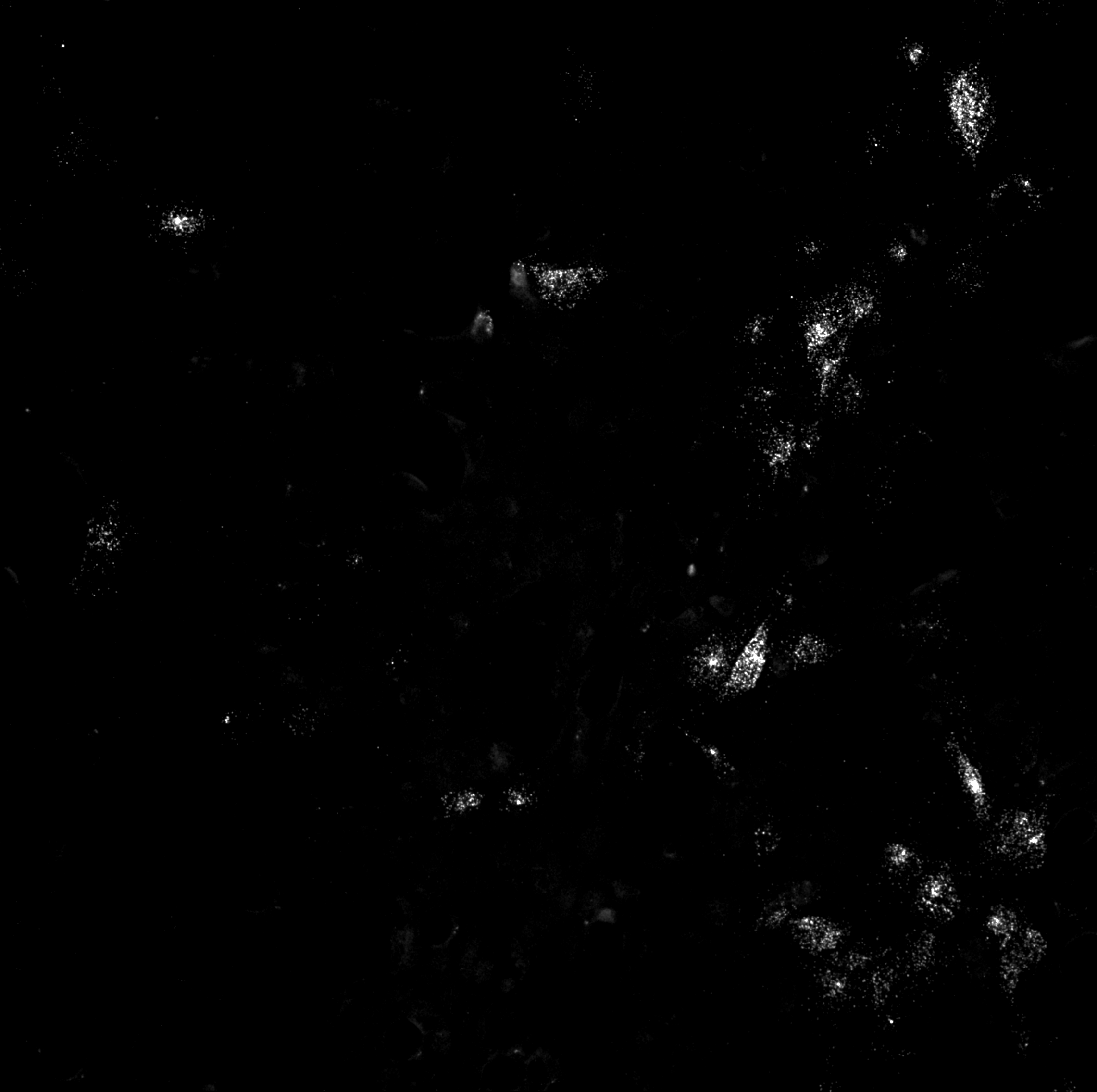

Supplement: Supplementary file 10 — Source Data for Figure 5 [file MSB-17-e9833-s001.zip › Figure 5/Figure 5A. 16hpi_FABP6(green) CCL2(red) DAPI(blue) AstV(white).tif]
